# Supplementary material for: K63 Ubiquitination of P21 Can Facilitate Pellino-1 in the Context of Chronic Obstructive Pulmonary Disease and Lung Cellular Senescence
Source: Cells. 2022 Oct 3;11(19):3115. doi: 10.3390/cells11193115 (PMC9563803; doi:10.3390/cells11193115)
Supplement: Supplementary file 1 [file cells-11-03115-s001.zip › Supplementary Figure and Tables.pdf]

*Supplementary Materials for*

# K63 Ubiquitination of P21 Can Facilitate Pellino-1 in the Context of Chronic Obstructive Pulmonary Disease and Lung Cellular Senescence

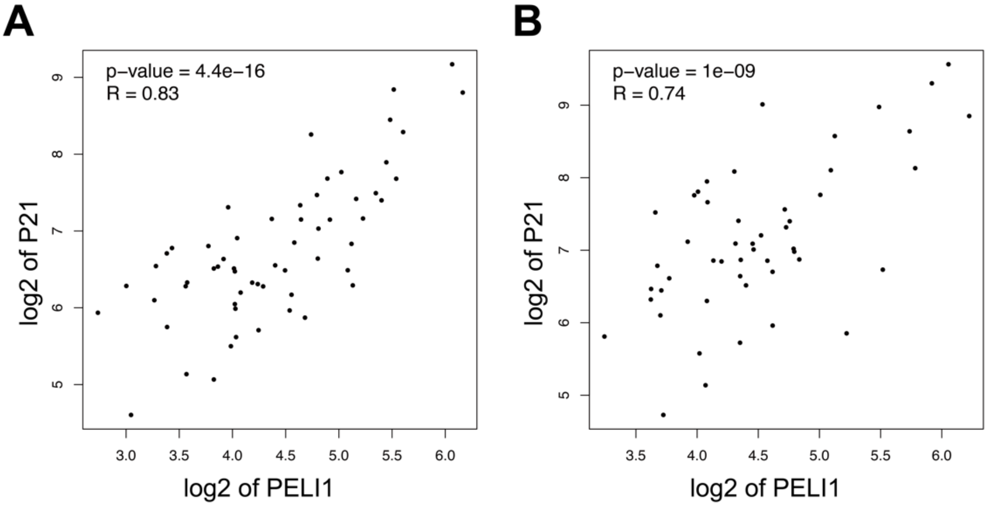


**Figure S1. Relationship between PELI1 and p21 in TCGA para-carcinoma lung tissue. Related to Figure 1.** (**A**) The relationship between PELI1 and p21 was significant in para-carcinoma lung tissue of the TCGA Lung adenocarcinoma (LUAD) database. (**B**) Relationship between PELI1 and p21 was significant in para-carcinoma lung tissue of TCGA Lung squamous cell carcinoma (LUSC) database. Data represent R for relative coefficient and P for significance.

**Table S1.** Primer for RT-qPCR.

| Primer Name | Sequence | Length | Tm |
|---|---|---|---|
| GAPDH Forward | CATGTACGTTGCTATCCAGGC | 21 | 60.8 |
| GAPDH Reverse | CTCCTTAATGTCACGCACGAT | 21 | 60.2 |
| Pellino-1 Forward | CAGCACTGTGCATATTGCTTG | 21 | 60.2 |
| Pellino-1 Reverse | CGGCCAATCTGAAACATATCGG | 22 | 61.4 |
| P21 Forward | TGTCCGTCAGAACCCATGC | 19 | 62.0 |
| P21 Reverse | AAAGTCGAAGTTCCATCGCTC | 21 | 60.4 |
| IL-6 Forward | ACTCACCTCTTCAGAACGAATTG | 23 | 60.2 |
| IL-6 Reverse | CCATCTTTGGAAGGTTCAGGTTG | 23 | 61.3 |
| IL-1$\alpha$ Forward | TGGTAGTAGCAACCAACGGGA | 21 | 62.9 |
| IL-1$\alpha$ Reverse | ACTTTGATTGAGGGCGTCATTC | 22 | 60.9 |
| MMP-9 Forward | TGTACCGCTATGGTTACACTCG | 22 | 61.5 |
| MMP-9 Reverse | GGCAGGGACAGTTGCTTCT | 19 | 61.9 |
| MMP-12 Forward | CATGAACCGTGAGGATGTTGA | 21 | 60.0 |
| MMP-12 Reverse | GCATGGGCTAGGATTCCACC | 20 | 62.4 |
| TNF-$\alpha$ Forward | TCCTGTGGAAGATCACCAATGT | 22 | 61.0 |
| TNF-$\alpha$ Reverse | GCAGGCACAACTTGTAGCC | 19 | 61.3 |
| CCL-2 Forward | CAGCCAGATGCAATCAATGCC | 21 | 62.3 |
| CCL-2 Reverse | TGGAATCCTGAACCCACTTCT | 21 | 60.4 |
| Cyclin E Forward | AAGGAGCGGGACACCATGA | 19 | 62.9 |
| Cyclin E Reverse | ACGGTCACGTTTGCCTTCC | 19 | 62.8 |

**Table S2.** Antibodies and Material Sources.

| REAGENT or RESOURCE | SOURCE | IDENTIFIER |
| --- | --- | --- |
| Antibodies | | |
| Rabbit anti-P21 (human and mouse) | Cell Signaling Technology (CST) | Cat# 37543; Cat# 2947 |
| Rabbit anti-P16 | CST | Cat# 18769 |
| Rabbit anti-Cyclin E1 | CST | Cat# 20808 |
| Rabbit anti-Rb | CST | Cat# 9313 |
| Rabbit anti-Phospho-Rb | CST | Cat# 8516 |
| Rabbit anti-P53 | CST | Cat# 2527 |
| Rabbit anti-Pellino-1 | CST | Cat# 31474 |
| Rabbit anti-SKP2 | CST | Cat# 2652 |
| Rabbit anti-GAPDH | CST | Cat# 2118 |
| Rabbit anti-HA | Abcam | Cat# ab236632 |
| Goat anti-Rabbit IgG Secondary Antibody, Western Blot | Invitrogen | Cat# 31466 |
| Goat anti-Rabbit IgG Secondary Antibody, Alexa Fluor 594 | Invitrogen | Cat# A-11012 |
| Goat anti-Rabbit IgG Secondary Antibody, Alexa Fluor 488 | Invitrogen | Cat# A-11008 |
| Chemicals, peptides, and recombinant proteins | | |
| Budesonide | Med Chem Express | Cat# HY-13580; CAS: 51333-22-3 |
| MG132 | Med Chem Express | Cat# HY-13259; CAS: 133407-82-6 |
| D-Galactose | Sigma-Aldrich | Cat# G5388; CAS: 59-23-4 |
| CSE (5%) | This paper | N/A |
| Critical commercial assays | | |
| Senescence β-Galactosidase Staining Kit | Beyotime, Shanghai, China | Cat# C0602 |
| Cell Cycle Analysis Kit | Beyotime, Shanghai, China | Cat# C1052 |
| IHC ABC stain kit | Zsbio, Beijing, China | Cat# PK-4002 |
| H&E stain kit | Beyotime, Shanghai, China | Cat# S0105 |
| Lipid Peroxidation MDA Assay Kit | Beyotime, Shanghai, China | Cat# S0131 |
| Total Superoxide Dismutase Assay Kit with NBT | Beyotime, Shanghai, China | Cat# S0109 |
| Mouse Tumor Necrosis Factor-$\alpha$ Enzyme-Linked Immuno-Sorbent Assay Kit | Beyotime, Shanghai, China | Cat# PT512 |
| Mouse Interleukin-6 Enzyme-Linked ImmunoSorbent Assay Kit | Beyotime, Shanghai, China | Cat# PI326 |
